# Supplementary figures and images for: Synergetic Effect of Tumor Treating Fields and Zinc Oxide Nanoparticles on Cell Apoptosis and Genotoxicity of Three Different Human Cancer Cell Lines
Source: Molecules. 2022 Jul 8;27(14):4384. doi: 10.3390/molecules27144384 (PMC9322763; doi:10.3390/molecules27144384)

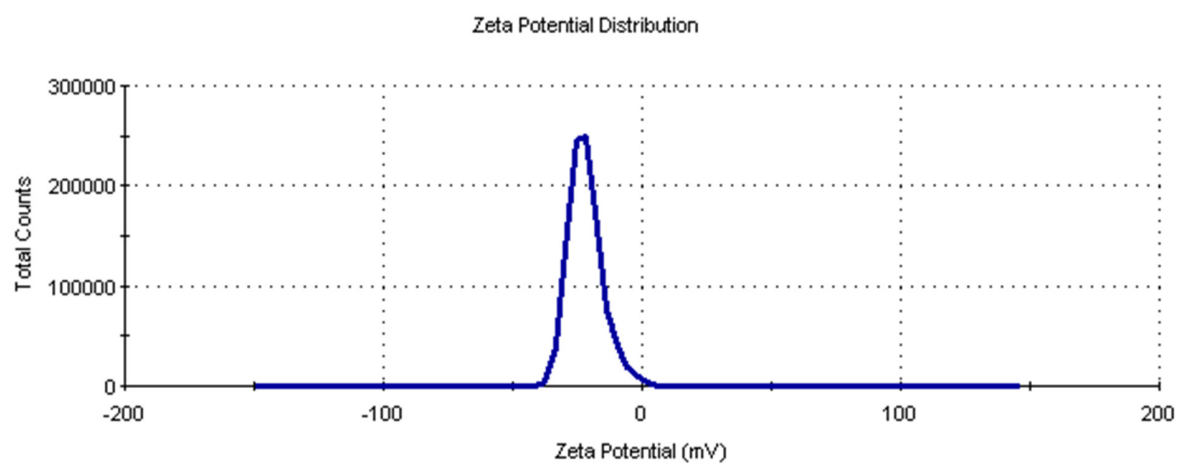

Figure S1. Zeta potential distribution of ZnO NPs.

Supplement: Supplementary file 1 [file molecules-27-04384-s001.zip › molecules-1798642-supplementary.pdf]
